# Extraction, Characterization, and In Vitro Biological Activity of Polyphenols from Discarded Young Fig Fruits Based on Deep Eutectic Solvents

**DOI:** 10.3390/antiox13091084

**Published:** 2024-09-04

**Authors:** Qinqiu Zhang, Yue Peng, Yi Xu, Fan Li, Shuxiang Liu, Danka Bukvicki, Qing Zhang, Shang Lin, Miaomiao Wang, Tianyi Zhang, Dingtao Wu, Wen Qin

**Affiliations:** 1 Sichuan Key Laboratory of Fruit and Vegetable Postharvest Physiology, College of Food Science, Sichuan Agricultural University, Ya’an 625014, China; 2020318064@stu.sicau.edu.cn (Q.Z.); pengyue2@stu.sicau.edu.cn (Y.P.); 13118177329@163.com (Y.X.); sliu@sicau.edu.cn (S.L.); zhangqing@sicau.edu.cn (Q.Z.); shanli@sicau.edu.cn (S.L.); wmm1871639054@163.com (M.W.); fupan351311@163.com (T.Z.); 2 Faculty of Biology, Institute of Botany 43, Belgrade University, 11000 Belgrade, Serbia; dankabukvicki@gmail.com; 3 Key Laboratory of Coarse Cereal Processing, Ministry of Agriculture and Rural Affairs, Sichuan Engineering and Technology Research Centre of Coarse Cereal Industrialization, School of Food and Biological Engineering, Chengdu University, Chengdu 610106, China; wudingtao@cdu.edu.cn

**Keywords:** utilization of waste resources, polyphenols, chlorogenic acid, antioxidant activity

## Abstract

(1) Background: Discarded young fig fruits (DYFFs) result in a waste of resources, such as sparse fruits and residual fruits, and there has been no research on the relationship between phenolic compounds and biological activity in DYFFs (2) Methods: Different deep eutectic solvents (DESs) and 80% ethanol were used to prepare DYFF extracts, and polyphenol extraction efficiency and bioactivities in the DYFFs extracts were compared. (3) Results: More than 1700 phytochemicals were identified in DYFFs, and thirteen of these typical phenolic compounds were analyzed quantitatively; chlorogenic acid, rutin, luteolin 8-C-glucoside, and epicatechin are the main polyphenols in DYFFs, especially chlorogenic acid with 2720–7980 mg/kg. Ferulic acid, caffeic acid, epicatechin, (+)-catechin, luteolin 8-C-glucoside, rutin, hesperetin, and chlorogenic acid showed different degrees of correlation with in vitro antioxidant activity. Moreover, the highest total phenol content found in the extracts of ChCl-Ethylene glycol (Choline chloride:Ethylene glycol = 1:2) was 8.88 mg GAE/g DW, and all quantitatively analyzed phenolic compounds had high levels in various DESs and 80% ethanol. The 80% ethanol and Choline chloride (ChCl) solvent system showed the greatest antioxidant properties, and the Choline chloride-Urea (Choline chloride: Urea = 1:2) extract of DYFFs exhibited the highest inhibitory activity. (4) Conclusions: DESs have demonstrated potential as promising green solvents, especially the ChCl solvent system, which facilitates the extraction of polyphenols.

## 1. Introduction

*Ficus carica* L. (FC) belongs to the genus *Ficus*, one of the oldest fruits grown in the Mediterranean region. Meanwhile, it also has high production in Mediterranean countries, especially Turkey. FC is rich in phytochemicals such as phenolics, organic acids, proteases, polysaccharides, and carotenoids [1]. All of the bioactive compounds in FC have health benefits such as antibacterial, antioxidant, and anti-diabetic properties, acetylcholinesterase inhibition, immune regulation, anti-cancer properties, etc. [2]. The powerful health benefits of FC are associated with their high levels and diversity of polyphenols, and polyphenols in FC are mainly phenolic acids, flavonoids, and anthocyanins [3]. 

FC contains phenolic acids such as chlorogenic acid, rutin, epicatechin, and many other polyphenols [4,5]. The types and content of polyphenols in figs at different ripening stages are completely different [6]; chlorogenic acid, ellagic acid, quercetin-3-O-rutinoside, quercetin-3-acetyl glucoside, anthocyanin-3-O-rutinoside, and anthocyanin-3-O-rutinoside were significantly different in unripe figs than in ripe figs [7]. Fruit thinning is a common practice employed to enhance the quality and yield of figs during the growing period, and some young fig fruits will be thinned and discarded; about 30–50% of unripe kiwifruits will be also thinned and wasted [8]. Currently, most of the studies are on mature fig fruits, and there are few studies on the discarded young fig fruits (DYFFs), such as sparse fruit and residual fruit, which is not conducive to the sustainable use of fig resources.

Moreover, deep eutectic solvents (DESs) are novel, green, and sustainable extractants with great potential for application in the extraction of plant polyphenols, polysaccharides, and other natural products [9]. DESs are mainly composed of hydrogen bond donors (HBDs) and acceptors (HBAs). The HBAs of DESs are mainly quaternary ammonium salt (such as choline chloride) or zwitterions (such as betaine) and the HBDs are mainly compounds such as urea, polyols, and sugars [10]. A ternary DES system (choline chloride: propionic acid: 1,3-butanediol) was used. Compared with the common traditional hot water extraction method, it resulted in 50% higher polysaccharide extraction and 70.4% lower protein content in the *Camellia oleifera* fruit shell [11]. Some studies have shown that the bioactivity of natural products extracted with deep eutectic solvents is significantly better than that of conventional solvent extraction [12]. A DES consisting of glycerol, xylitol, and D-(-)-fructose in a 3:3:3 molar ratio showed higher extraction rates for the five polyphenols simultaneously compared to conventional ethanol solvents in leaves of FC, and the extraction rates of caffeoyl malic acid, glucoside psoralen, rutin, psoralen, and bergamot lactone were 6.48 mg/g, 16.34 mg/g, 5.21 mg/g, 15.22 mg/g, and 2.46 mg/g, respectively [13]. There is also no study on the extraction of polyphenols from DYFFs by DESs.

This study evaluated the extraction efficiency, composition, and biological activities of 80% ethanol solvents and DESs for polyphenols present in discarded young fig fruits (DYFFs). The biological activities of the extracts were analyzed, mainly including antioxidant, pancreatic lipase, and α-amylase inhibition activities. Moreover, the efficiency of various solvents in extracting polyphenols and the biological activities of DYFFs were investigated by multivariate analysis, including principal component analysis (PCA), hierarchical cluster analysis and Pearson’s correlation analysis. 

## 2. Materials and Methods

### 2.1. Materials and Chemicals

The discarded young fig fruits (Brunswick *Ficus carica L.*, unpeeled) utilized in this study were procured in July 2023 from Nei-Jiang, Sichuan Province, China. After being sliced and frozen at −80 °C, the sample was subjected to vacuum freeze drying for 36–48 h; after that, it was crushed into powder and extracted by various solvents. All standards, mainly including polyphenols, were sourced from Sigma-Aldrich (Shanghai, China), which also provided the pancreatic lipase (USP*4) and the p-nitrophenyl laurate (pNP-laurate) required. All other chemicals were purchased from China Aladdin Co., LTD, Wuhan, China.

### 2.2. Extraction Methods

A total of 1 g of lyophilized DYFF powder was added to 20 mL of extraction solvent (either traditional solvent 80% ethanol or DESs) and subjected to 20–30 min ultrasonic extraction at 400 W and 40 °C. The mixtures were then centrifuged at 6000× *g* for 15 min, and finally, supernatants were collected and stored at 4 °C.

### 2.3. The Composition of DESs

Wang et al.’s method was used to select and prepare 13 DESs [9]. The DES components were accurately weighed in a specific ratio mixed (Table 1) with 30% water (*w*/*w*), heated at 80 °C using a magnetic stirrer with continuous stirring, and then cooled to room temperature to obtain the DESs (the uncontrolled pH of these DESs).

### 2.4. Determination of Total Phenols and Total Flavonoids 

Previous methods were used with slight modification [14,15]. A total of 100 µL of DYFF extracts or standards at different dilutions was mixed with 100 µL of Folin–Ciocalteu. Then, the mixtures were reacted at room temperature in the dark, then 1000 µL of Na_2_CO_3_ solution was added and incubation was continued at 25 °C, in a dark environment for 30 min (the blank was made with 80% ethanol instead of the sample, and the subsequent operations were the same). Finally, the absorbance was measured at 765 nm. TPC was expressed as gallic acid equivalents (GAE) per gram of dry weight of DYFFs (mg GAE/g DW).

A total of 200 μL rutin standard solution (or 200 μL of sample dilution solution) was added into a 5mL EP tube, and 120 μL of 5% NaNO_2_ solution was added, shaken, and put at 25 °C for 6 min. Then, 120 μL of 10% Al (NO_3_)_3_ solution was added, shaken well, and left to stand for 6 min, and then 1600 μL of NaOH solution with a concentration of 4% was added, mixed thoroughly, and then reacted for 15 min at 25 °C. Finally, the solution was measured at 510 nm, with TFC expressed in rutin equivalent (mg RE/g DW).

### 2.5. Identification of Polyphenols from DYFFs by UPLC/ESI-TRAP-MS/MS

#### 2.5.1. Sample Preparation and Extraction

The fig extract was removed from the −80 °C refrigerator, thawed until there was no ice in the sample, vortexed for 10 s, and mixed well. A total of 100 μL of the sample was added to the corresponding numbered 1.5 mL centrifuge tube, and 100 μL of 70% methanol with internal standard extraction solution (less than 100 μL, add the extraction solution at a ratio of 1:1 (*v*/*v*)) was added. It was then vortexed for 15 min at 12,000 r/min at 4 °C and centrifuged for 3 min. Supernatants were removed, filtered through a microporous membrane (0.22 µm pore size) and stored in injection vials for LC-MS/MS detection.

#### 2.5.2. Detection Conditions

UPLC was used to analyze the diluted DYFF extracts through the ESI -MS/MS system. The following analytical conditions were used in the experimental setup: Column, Agilent SB-C18(1.8 µm, 2.1 mm × 100 mm) and the column oven was set to 40 °C. Mobile phases, Solvent A (0.1% formic acid) and Solvent B (acetonitrile containing 0.1% formic acid), were used for the sample measurements, with 95% A, 5% B as the starting conditions, which were adjusted to 5% A and 95% B within 9 min, and held for 1 min. Subsequently, the mobile phase was adjusted to a solution of 95% A and 5.0% B and held for 2.9 min. The flow rate was set to 0.35 mL/min. The effluent was then connected to an ESI-triple quadrupole linear ion trap (QTRAP)-mass spectrometer. Moreover, refer to Chen et al.’s method for modification [16,17]. The following parameters were employed in the ESI source operation: the source temperature was 550 °C; the ion spray voltage was 5500 V (positive mode) and −4500 V (negative mode); and ion source gas I, gas II, and curtain gas were set to 50, 60, and 25 psi, respectively, with a high collision activation dissociation value. A QQQ scan was acquired as a multiple reaction monitoring (MRM) experiment with nitrogen as a collision gas. 

### 2.6. Quantification of DYFFs by HPLC-DAD

The samples were pre-treated and determined by HPLC-DAD. The chromatographic column was Acclaim TM beat C18 (3.5 μm, 250 mm × 4.6 mm) with mobile phase: A (0.1% formic acid in water) and B (acetonitrile); column temperature: 30 °C; flow rate: 0.3 mL/min; and injection volume: 5 μL. A gradient elution of A and B was used as the mobile phases: 0-5 min, 15% A; 5–25 min, 25–35% A; 25–40 min, 35–50% A; 40–45 min, 85% A; and 45–50 min, 15% A. The contents of the identified polyphenols in the DYFFs were calculated from the standard curves of the corresponding standards [18].

### 2.7. Assessment of the Antioxidant Capacity

Refer to the method of Nie et al. and modify it lightly [19]. DPPH and ABTS^·+^ scavenging assays were used to determine the free radical scavenging capacity of noni fruit extracts expressed as water-soluble vitamin E equivalents (Trolox equivalents), per gram dry weight of DYFFs (mg Trolox/g DW). A total of 100 μL of fig extract or Trolox at the appropriate dilution was incubated with 800 μL of 100 μmol/L DPPH-methanol solution in the dark at 25 °C for 30 min to allow complete reaction, and measured at 517 nm.

A total of 7 mmol/L ABTS^·+^ solution was mixed with 2.45 mmol/L K_2_S_2_O_8_ solution in equal volumes and incubated at 25 °C in the dark for 12–16 h, then incubated at 517 nm for 30 min. The absorbance of the freshly prepared ABTS^·+^ primary solution was adjusted to 0.70 ± 0.02 at 734 nm with distilled water. A total of 100 μL of fig fruit extract or Trolox at appropriate dilutions was mixed with 800 μL of freshly diluted ABTS^·+^ solution and then incubated for 30 min at 25 °C, and all process reactions needed to take place in the dark, finally measured at 734 nm.

Reducing power, this involved a slight modification of the method of Guo et al. [20]. A total of 200 µL of 10% K_3_[Fe (CN)_6_] and 100 µL of different concentrations of samples were well mixed. A total of 100 µL of 10% trichloroacetic acid was added after a 20 min reaction at 50 °C and protected from light. Then, 200 µL of ultrapure water and 40 µL of 0.1% FeCl_3_ were added and reacted for 30 min at 50 °C, and the OD value was measured at 700 nm, using Trolox as the standard and expressed as mg Trolox/g DW.

Ferric reducing antioxidant power assay (FRAP), and the FRAP reagent was synthesized by combining acetate buffer (300 mmol/L, pH = 3.6), 10 mmol/L tripyridyltriazine, and 20 mM FeCl_3_ in a 10:1:1 ratio, and using a slight modification of the method of Yuan et al. [21]. A total of 100 µL of different concentrations of DYFF extract solution was mixed with 3 mL FRAP. The reaction was carried out at room temperature, in the dark, for 4 min, and the OD value was read at 593 nm; it was also expressed as mg Trolox/g DW.

### 2.8. Pancreatic Lipase and α-Amylase Activity Inhibition Assay

Pancreatic lipase inhibitory activity was determined according to the method of Wang et al. and revised [9]. pNP-laurate (0.08%, *w*/*v*) was dissolved in 5 mmol/L sodium acetate and heated to dissolve. A total of 100 uL of various extracts of DYFFs (or orlistat) was mixed with 600 µL of 5 mg/mL of pancreatic lipase and incubated at 37 °C for 10–20 min; then, 1800 μL of pNP -laurate was added, incubated at 37 °C for 60–120 min, and measured at 405 nm, and the result was expressed as mg orlistat equivalents/mL (ug OTT/mL).

The determination of the inhibition of a-amylase activity was based on the previous literature method [22]. Briefly, 100 μL of diluted DYFF extracts or acarbose were mixed with 100 μL of a-amylase (1.25 U/mL) solution and incubated at 37 °C for 10 min. A total of 200 μL soluble starch solution (1%, *w*/*v*) was added and incubated at 37 °C for 10 min, 800 μL of DNS reagent was added to terminate the reaction, and the solution was heated at 95 °C for 10 min, then diluted with pure water. Finally, it was measured at 540 nm and the result was expressed as mg acarbose equivalents/mL (mg AE/mL).

### 2.9. Statistical Analysis

Quantitative data were analyzed by ANOVA combined with Duncan’s test using SPSS 26 software (IBM, Armonk, NY, USA), with differences statistically significant at *p* < 0.05. All other statistical graphs were performed using Graph pad Prism 9 software and Originpro2024.

## 3. Results and Discussion

### 3.1. Quantification of Polyphenols from DYFFs by UPLC/ESI-Q TRAP-MS/MS

The traditional solvent extraction method is the most conventional method for the extraction of plant-derived polyphenols. Accordingly, the extract of DYFFs obtained through the use of 80% ethanol was selected for the analysis and identification of its phytochemical composition through the application of UPLC/ESI-Q TRAP-MS/MS. More than 1700 phytochemicals were identified by the fragmentation information of the parent ions and secondary mass spectrometry in combination with mass spectrometry databases for comparison and identification [23].

The main polyphenols are listed in Table 2 and Appendix A. There are 11 kinds of phenolic acids, including cryptochlorogenic acid, chlorogenic acid, neochlorogenic acid, coniferyl ferulate, isoferulic acid, gallic acid-1-O-xyloside, grevilloside F, ferulic acid-4-O-glucoside, ferulic acid, salicylic acid-2-O-glucoside, and caffeic acid. There are 33 kinds of flavonoids, mainly including rutin, quercetin-7-O-rutinoside, catechin, epicatechin, apigenin-6-C-(2″-glucosyl) arabinoside, kaempferol-3-O-neohesperidoside, luteolin-8-C-glucoside, kaempferol-3-O-glucoside-7-O-rhamnoside, apigenin-6-C-glucoside, luteolin-6-C-glucoside, etc. Moreover, 11 major kinds of lignans and coumarins were found, such as syringaresinol-4-O-β-D-glucopyranoside, coumarin, psoralen, and pinoresinol.

### 3.2. Effects of Different DESs on Polyphenols in DYFFs

The quantitative analysis of 13 representative polyphenols in the extracts of DYFFs with different deep eutectic solvents and 80% ethanol using HPLC-PDA can reveal the differences in the extraction ability and selectivity of solvent types for different phenolic fractions, and the results are shown in Table 3 and Table 4. For five phenolic acids (gallic acid, salicylic acid, chlorogenic acid, caffeic acid, and ferulic acid) and eight flavonoids (rutin, (+)-catechin, epicatechin, kaempferol, quercetin, luteolin-8-C-glucoside, apigenin, and hesperidin). The HPLC-PDA results showed significant differences in the extraction efficiency of these polyphenols with different extraction solvents. Gallic acid, kaempferol, quercetin, salicylic acid, and apigenin were not detected; this may be because the relative content is too low to allow quantitative analysis. Chlorogenic acid, rutin, luteolin 8-C-glucoside, and epicatechin are the main polyphenols in DYFFs; the chlorogenic acid was significantly higher than others, it could attach 2720–7980 mg/kg, and rutin followed it at 456–943 mg/kg (*p* < 0.05). ChCl-TA, ChCl-LaA, and 80% ethanol had the highest extraction efficiency for chlorogenic acid, ChCl-MaA and Bet-MaA-Glu followed, and Pro-EtG had the lowest extraction efficiency for chlorogenic acid, with only 2720 ± 150 mg/kg DW (*p* < 0.05). However, 80% ethanol showed the highest extraction efficiency for epicatechin, luteolin 8-C-glucoside, rutin, hesperetin, caffeic acid, and ferulic acid. Moreover, compared to the Choline chloride (ChCl) solvent system, 80% ethanol, MA-EG, the Bet-solvent system, and the pro-solvent system showed the lowest extraction efficiency for (+)-catechin and epicatechin. The trends of these findings were similar to those of Wang et al. in that DESs are very promising extraction solvents, especially the ChCl solvent system, for the selective extraction of phenolic acids from DYFFs [24]. 

### 3.3. Effects of Different DESs on Total Phenols and Total Flavonoids in DYFFs

**Figure 1:**
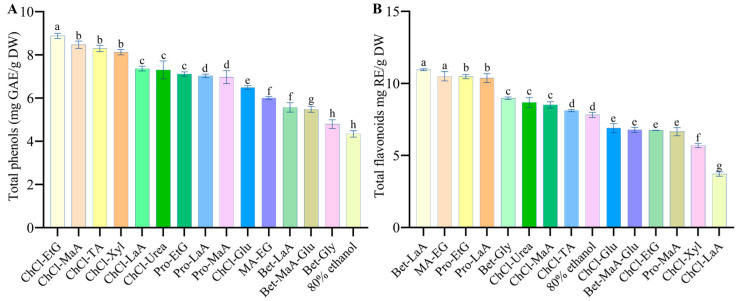
Effect of different extraction solvents on total phenols content in DYFFs (**A**) and total flavonoids content (**B**); a, b, c, etc., indicate differences (*p* < 0.05), and the results are expressed as the mean ± standard deviation of three values.

The TPC and TFC of DYFFs varied according to the solvents (13 different DESs and 80% ethanol), as shown in Figure 1. The total phenols content varied from 4.34 to 8.88 mg GAE/g DW, and the total flavonoids content varied from 3.71 to 10.95 mg QE/g DW. The highest total phenols content was found in ChCl-EtG, ChCl-MaA, and ChCl-TA extracts, and the lowest total phenols content was found in the 80% ethanol extract (*p* < 0.05). This is similar to the results and conclusions of Fu et al. where the ChCl solvent system had the highest total phenols extractions [25].

Meanwhile, the highest total flavonoids were in the Bet-LaA extract, followed by MA-EG and Pro-EtG, and the lowest total flavonoids were in the ChCl-LaA extract (*p* < 0.05). In terms of total phenols, the solvent extraction efficiencies measured were, in descending order, as follows: ChCl-EtG, ChCl-MaA, ChCl-TA, ChCl-Xyl, ChCl-LaA, ChCl-Urea, Pro-EtG, Pro-LaA, Pro-MaA, ChCl-Glu, MA-EG, Bet-LaA, Bet-MaA-Glu, Bet-Gly, and 80% ethanol. In terms of total flavonoids, the solvent extraction efficiencies measured were, in descending order, as follows: Bet-LaA, MA-EG, Pro-EtG, Pro-LaA, Bet-Gly, ChCl-Urea, ChCl-MaA, ChCl-TA, 80% ethanol, ChCl-Glu, Bet-MaA-Glu, ChCl-EtG, Pro-MaA, ChCl-Xyl, and ChCl-LaA (*p* < 0.05).

### 3.4. Effects of Different Deep Eutectic Solvents on Biological Activities In Vitro from DYFFs

**Figure 2:**
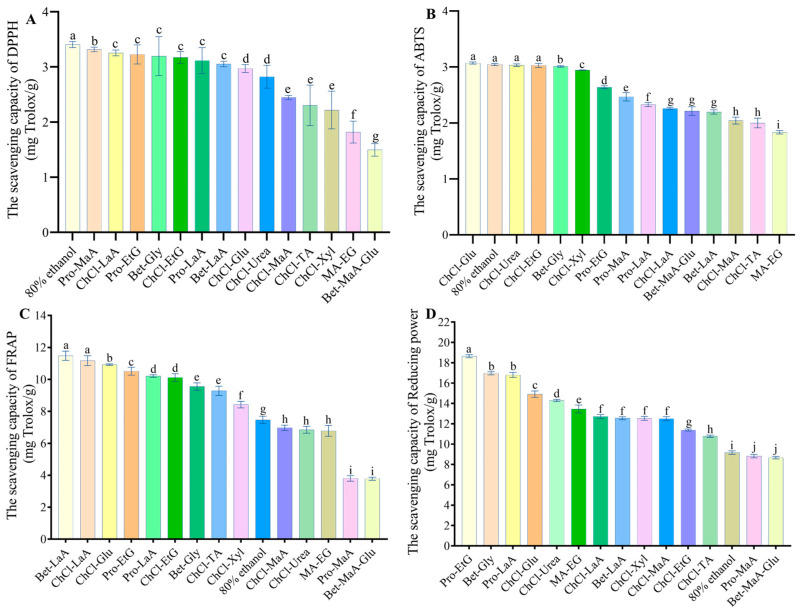
Effect of different extraction solvents on DPPH radical scavenging rate (**A**), ABTS radical scavenging rate (**B**), FRAP activity (**C**), and reducing power (**D**); a, b, c, etc., indicate a significant difference (*p* < 0.05), and the results are expressed as the mean ± standard deviation of three values.

The antioxidant, pancreatic lipase, and a-amylase inhibitory activities of DYFFs extracted from different DESs were determined, and the results are presented in Figure 2 and Table 5. All DES extracts showed excellent antioxidant properties, and 80% ethanol extracts of DYFFs showed the best DPPH and ABTS radical scavenging capacity, 3.41 ± 0.06 mg Trolox/g and 3.07 ± 0.02 mg Trolox/g, respectively. This is consistent with the findings of Wang et al. that ethanol solvent has the best antioxidant capacity as an extraction solvent [9]. ChCl-Glu, ChCl-Urea, and ChCl-EtG also showed the best ABTS radical scavenging capacity of 3.05 ± 0.02, 3.03 ± 0.02, and 3.028 ± 0.04 mg Trolox/g, respectively, whereas MA-EG was the lowest at 1.84 ± 0.031 mg TROLOX/g (*p* < 0.05). Bet-LaA and ChCl-LaA showed the strongest FRAP activity of 11.49 ± 0.29 and 11.17 ± 0.31 mg Trolox/g, respectively (*p* < 0.05). Pro-EtG showed the strongest total reducing power (RP) of 18.64 ± 0.15 mg Trolox/g. Meanwhile, Bet-MaA-Glu, except for ABTS, showed the worst antioxidant in DPPH radical scavenging capacity, ABTS radical clearing capacity, and FRAP activity (*p* < 0.05); this is related to the fact that it contains lower levels of TPC and TFC.

Furthermore, the higher the equivalent of the standard (orlistat and acarbose), the stronger the ability to inhibit the enzyme activity. The ChCl-Urea extract of DYFFs exhibited the highest inhibitory activity against α-amylase at 5.87 ± 0.80 mg acarbose equivalents (AE)/mL, followed by ChCl-LaA and ChCl-MaA at 3.54 ± 0.23 mg AE/mL and 2.93 ± 0.31 mg AE/mL, respectively. Bet- MaA-Glu and Pro-MaA showed the lowest inhibitory activity of only 0.024 ± 0.004 mg AE/mL and 0.015 ± 0.003mg AE/mL (*p* < 0.05), and this suggests fig may also be a natural source of diabetes relievers. Phenolic acids are also involved in modulating the activity of starch digestive enzymes, and they inhibit α-amylase and α-glucosidase activities [26]. The Bet-MaA-Glu and ChCl-LaA extracts of DYFFs exhibited the highest pancreatic lipase inhibitory activity of 6010 ± 168 and 5810 ± 483 ug OTT/mL, respectively. The 80% ethanol extracts showed the lowest inhibitory activity of only 414 ± 22 ug OTT/mL. The pancreatic lipase inhibitory activity of all DES extracts was significantly higher than that of 80% ethanol (*p* < 0.05); it was also in accord with the findings of Wang et al. and Herrera et al. [9,27]. However, the extracts are mixtures that may contain multiple bioactive substances that inhibit lipase activity; it is necessary to further analyze the relationship between the bioactive substances in DYFFs extracts and the inhibitory activity of pancreatic lipase [28].

### 3.5. Difference Analysis

Multivariate statistical analysis is commonly used as a data analysis tool to provide an overall description of a data set. PCA is often used to reduce the multidimensional structure of the data and provide plots for interpreting the variance [29]. 

**Figure 3:**
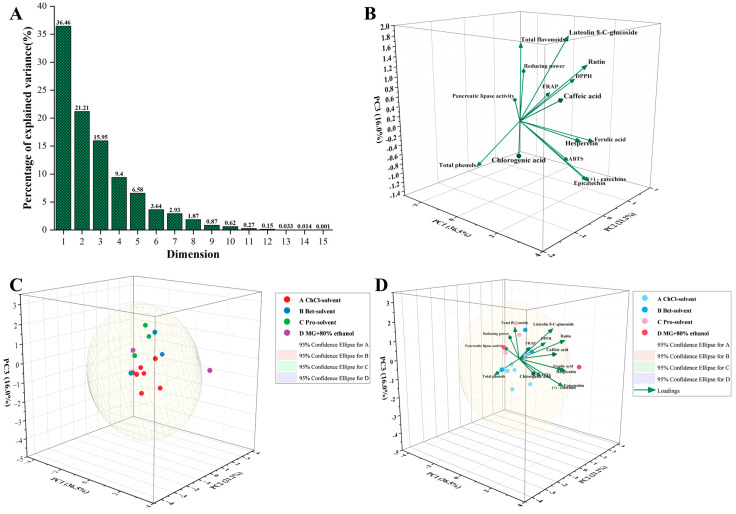
Scree plot of PCA for the percentage of explained variance (**A**). Loading plot of polyphenols and bioactivities based on PCA (**B**). Scores plot of different extraction solvent systems based on PCA (**C**). Biplot of PCA (**D**).

PCA has been widely used to analyze the differences between active ingredients and biological activities in extracts with different solvents [30]. In order to make a better comprehensive assessment and reduce the overlap between the information of the indicators, α-amylase inhibition activity, pancreatic lipase activity, DPPH radical scavenging rate, ABTS radical scavenging rate, FRAP activity, reducing power, total phenols content, total flavonoids content, hesperetin, caffeic acid, chlorogenic acid, luteolin 8-C-glucoside, rutin, (+)-catechin, epicatechin, and ferulic acid, 16 indicators were selected to establish a matrix, and then a PCA analysis of the 16 indicators was performed using Origin lab 2024. The scree plot shows the first three dimensions of the principal components (Figure 3A), explaining 73.61% of the total variance, with the first dimension explaining 36.46% of the total variance, the second dimension explaining 21.21% of the total variance, and the third dimension explaining 15.95% of the total variance. Based on the loading diagram it can be found that the major contributors to the first dimension were hesperetin (40.2%), rutin (39.94%), caffeic acid (37.36%), and (+)-catechin (31.89%). Contributing mainly to the second dimension were ABTS radical scavenging activity (41.72), DPPH radical scavenging activity (39.36%), total reducing power (32.89%), and FRAP activity (29.70%). Greater contributions to the third dimension were luteolin 8-C-glucoside (46.74%), total flavonoids (39.03%), and rutin (32.84%). A loadings plot shows which variables positively and negatively correlate with the principal components. Positive loadings indicate that the variable varies in the same direction as the principal component. In contrast, negative loadings indicate that the variable varies in the opposite direction of the principal component [31]. As shown in Figure 3B, the indicators are close to each other, indicating a high positive correlation, and FRAP activity, DPPH radical scavenging capacity, caffeic acid, luteolin 8-C-glucoside, and rutin were close to each other, which indicated that caffeic acid, luteolin 8-C-glucoside, and rutin made positive contributions to FRAP activity and DPPH radical scavenging capacity. Total flavonoids, pancreatic lipase activity, and total reducing power were close to each other, and total flavonoids positively contributed to total reducing power and pancreatic lipase activity. Similarly, the positive contribution of hickory bushel polyphenols and baobab shell polyphenols to antioxidant activities including DPPH radical scavenging activity, ABTS radical scavenging activity, FRAP activity, and total reducing power was reported by Fu et al. and Ismail et al. [25,32].

In the score plot of different solvents (Figure 3C and Table 6), each point represents a solvent extract, and its coordinate value on PC1, PC2, and PC3 indicates the score of that solvent extract on these three principal components, and the position of the point reflects the relative position of the solvent extract in the principal component space. The major contributors to the first dimension were 80% ethanol (302.76%), ChCl-LaA (85.56%), ChCl-EtG (42.42%), and Bet-LaA (36.25%) in that order. The remaining factors showed mostly negative contributions. The factors contributing significantly to the second dimension were Bet-Gly (173.95%), Pro-EtG (141.05%), 80%ethanol (115.56%), ChCl-Glu (105.18%), ChCl-EtG (44.12%), and ChCl-Urea (19.86%). The factors contributing significantly to the third dimension contribution were Bet-LaA (157.33%), Pro-LaA (184.56%), Pro-EtG (91.12%), MA-EG (72.17%), and Pro-MaA (39.10%). In summary, in Figure 3D, the extracts using the ChCl-solvent system and 80% ethanol achieved higher values in the first and second dimensions, mainly from the indexes including hesperetin, caffeic acid, rutin, (+)-catechin, ABTS radical scavenging activity, DPPH radical scavenging activity, total reducing power, and FRAP activity. Meanwhile, Zhou et al. extracted polyphenols from *Morus alba L.* leaves using DESs and investigated their extraction efficiency using principal component analysis, and they obtained similar results; ChCl solvent system for phenolics extraction had a high extraction efficiency and wider efficient extraction range [33,34,35].

**Figure 4:**
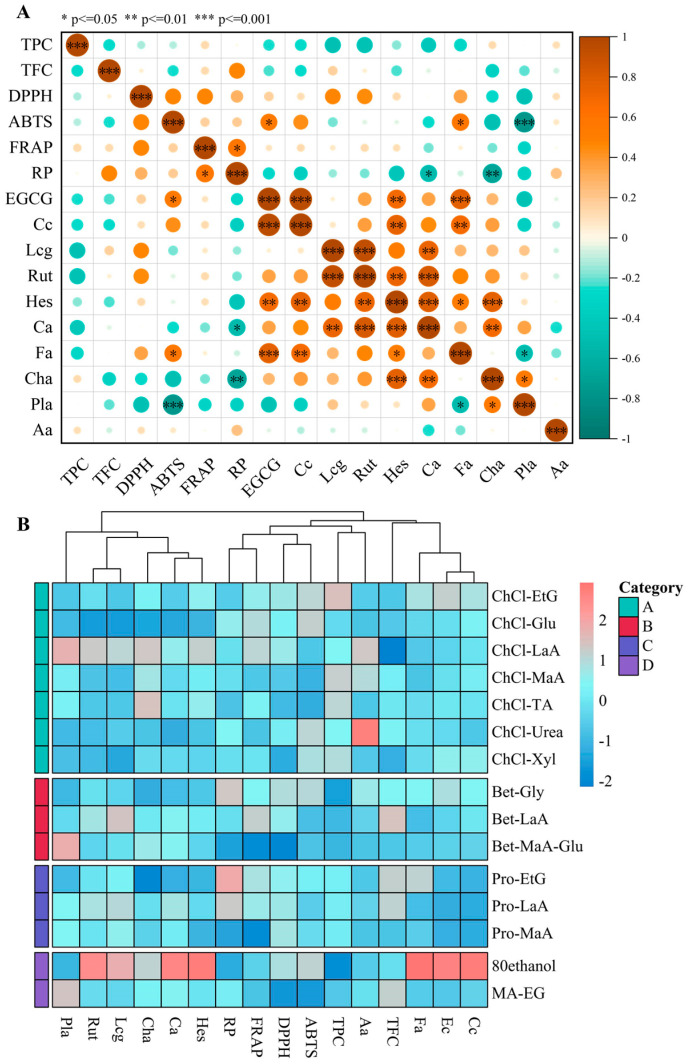
Correlation coefficients between polyphenols and bioactivities (Pearson correlation coefficient, *p* < 0.05) (**A**). Heatmap with cluster analysis of extracts according to polyphenols and bioactivities (**B**). Aa: α-amylase inhibition activity; Pla: pancreatic lipase activity; DPPH: DPPH radical scavenging rate; ABTS: ABTS radical scavenging rate; FRAP: FRAP activity; RP: Reducing power; TPC: total phenols content; TFC: total flavonoids content; Hes: hesperetin; Ca: caffeic acid; Cha: chlorogenic acid; Lcg: luteolin 8-C-glucoside; Rut: rutin; Cc: (+)-catechin; Ec: epicatechin; Fa: ferulic acid.

The correlation analysis of polyphenols in DYFF extracts with their in vitro bioactivities is shown in Figure 4A. It can be seen that ferulic acid, caffeic acid, epicatechin, (+)-catechin, luteolin 8-C-glucoside, rutin, hesperetin, and chlorogenic acid showed different degrees of correlation with in vitro antioxidant activity, especially ferulic acid, caffeic acid, epicatechin, and catechin, showed significant positive correlation with in vitro antioxidant properties. Ferulic acid, chlorogenic acid, and ABTS radical scavenging activity also exhibited a remarkable correlation with pancreatic lipase inhibition activity. Furthermore, the extracts prepared with the different solvents were easily distinguished and mainly classified into four categories, which were labeled “A”, “B”, “C”, and “D”. The extracts prepared with the ChCl solvent system were grouped into cluster A, the extracts obtained by the Bet solvent system were mainly grouped into cluster B, the extracts obtained by the Pro solvent system mainly grouped into cluster C, and the extract obtained by 80% ethanol and MA-EG formed cluster D [36]. The heat map based on a cluster analysis of polyphenols with in vitro activity directly demonstrated the effect of different solvents on each index (Figure 4B). Based on the results of cluster analysis, the extracts using different solvents were classified into four categories. The ChCl-solvent system and ethanol solvent showed better extraction of DYFFs, while the extracts exhibited better in vitro activity, a result that was also similar to that in the PCA analysis. Collectively, the findings suggest that the ChCl-solvent system can be applied as an efficient technique for the preparation of bioactive polyphenolics from DYFFs, and it also can improve the potential of DYFFs to be developed and utilized as functional foods and nutraceuticals.

## 4. Conclusions

In this study, more than 1700 phytochemicals were detected in DYFF extracts through UPLC/ESI-Q TRAP-MS/MS, among which polyphenols mainly included phenolic acids, flavonoids, coumarins, and lignan substances. Thirteen of these typical polyphenols were analyzed quantitatively, chlorogenic acid, rutin, luteolin 8-C-glucoside, and epicatechin are the main polyphenols in DYFFs, especially chlorogenic acid, which is 2720–7980 mg/kg. Moreover, the highest TPC was found in the extracts of ChCl-EtG, all quantitatively analyzed polyphenols had high levels in various DESs and 80% ethanol. The 80% ethanol and ChCl solvent system showed the best antioxidant properties, and the ChCl-Urea extract of DYFFs exhibited the highest inhibitory activity. DESs have shown potential as promising green solvents, especially the ChCl solvent system, which facilitates the extraction of fig polyphenols.

## Figures and Tables

**Table 1:** Different DES compositions.

DES Compositions	Abbreviation	Molar Ratio
Choline chloride: malic acid	ChCl-MaA	1.5:1
Choline chloride: tartaric acid	ChCl-TA	1.5:1
Choline chloride: xylitol	ChCl-Xyl	1:1
Choline chloride: ethylene glycol	ChCl-EtG	1:2
Choline chloride: urea	ChCl-Urea	1:2
Choline chloride: lactic acid	ChCl-LaA	1:2
Choline chloride: glucose	ChCl-Glu	2:1
Betaine: malic acid: glucose	Bet-MaA-Glu	1:1:1
Betaine: ethylene glycol	Bet-Gly	1:2
Betaine: lactic acid	Bet-LaA	1:2
Malic acid: ethylene glycol	MA-EG	1:2
L-proline: lactic acid	Pro-LaA	1:2
L-proline: ethylene glycol	Pro-EtG	1:2
L-proline: malic acid	Pro-MaA	1:1

**Table 2:** Identification of the main polyphenols in DYFFs.

Compounds	Molecular Weight (Da)	Formula	Ionization Model	Class I	Ion Current Intensity
Cryptochlorogenic acid	354.10	C_16_H_18_O_9_	[M-H]^−^	Phenolic acids	70,985,822.20
Chlorogenic acid	354.10	C_16_H_18_O_9_	[M-H]^−^	Phenolic acids	65,833,453.80
Neochlorogenic acid	354.10	C_16_H_18_O_9_	[M+H]^+^	Phenolic acids	46,456,076.33
Coniferyl ferulate	356.13	C_20_H_20_O_6_	[M-H]^−^	Phenolic acids	28,323,336.86
Isoferulic Acid	194.06	C_10_H_10_O_4_	[M-H]^−^	Phenolic acids	15,431,160.68
Gallic acid-1-O-xyloside	302.06	C_12_H_14_O_9_	[M-H]^−^	Phenolic acids	11,456,334.24
Grevilloside F	342.10	C_15_H_18_O_9_	[M-H]^−^	Phenolic acids	10,661,505.14
Ferulic acid-4-O-glucoside	356.11	C_16_H_20_O_9_	[M-H]^−^	Phenolic acids	10,078,917.79
Ferulic acid	194.06	C_10_H_10_O_4_	[M-H]^−^	Phenolic acids	8,443,288.36
Salicylic acid-2-O-glucoside	300.08	C_13_H_16_O_8_	[M-H]^−^	Phenolic acids	7,957,267.37
Caffeic acid	180.04	C_9_H_8_O_4_	[M-H]^−^	Phenolic acids	4,424,501.55
Quercetin-3-O-robinobioside	610.15	C_27_H_30_O_16_	[M-H]^−^	Flavonoids	56,406,063.27
Rutin	610.15	C_27_H_30_O_16_	[M+H]^+^	Flavonoids	45,322,065.47
Rutin Trihydrate	664.19	C_27_H_36_O_19_	[M-3H_2_O+H]^+^	Flavonoids	43,811,430.23
Quercetin-7-O-(6″-malonyl) glucoside	550.10	C_24_H_22_O_15_	[M+H]^+^	Flavonoids	42,809,212.80
Quercetin-7-O-rutinoside	610.15	C_27_H_30_O_16_	[M+H]^+^	Flavonoids	42,608,141.44
Quercetin-3-O-neohesperidoside	610.15	C_27_H_30_O_16_	[M+H]^+^	Flavonoids	42,337,008.56
Quercetin-3-O-glucoside-7-O-rhamnoside	610.15	C_27_H_30_O_16_	[M+H]^+^	Flavonoids	41,808,386.35
Quercetin-3-O-(4″-O-glucosyl) rhamnoside	610.15	C_27_H_30_O_16_	[M+H]^+^	Flavonoids	41,352,467.53
Licoagrochalcone D	354.15	C_21_H_22_O_5_	[M+H]^+^	Flavonoids	38,377,224.37
Licoflavonol	354.11	C_20_H_18_O_6_	[M+H]^+^	Flavonoids	29,038,302.51
Isoquercitrin	464.10	C_21_H_20_O_12_	[M+H]^+^	Flavonoids	28,073,065.84
Quercetin-3-O-alloside; Isohyperoside	464.10	C_21_H_20_O_12_	[M+H]^+^	Flavonoids	28,014,100.57
Hesperetin-3’-O-glucoside	464.13	C_22_H_24_O_11_	[M+H]^+^	Flavonoids	25,911,462.31
Quercetin-3-O-galactoside	464.10	C_21_H_20_O_12_	[M-H]-	Flavonoids	24,277,337.93
Quercetin-5-O-β-D-glucoside	464.10	C_21_H_20_O_12_	[M+H]^+^	Flavonoids	19,112,040.60
Quercetin-3-O-(6″-O-acetyl) galactoside	506.11	C_23_H_22_O_13_	[M-H]^−^	Flavonoids	17,249,778.81
Catechin	290.08	C_15_H_14_O_6_	[M+H]^+^	Flavonoids	16,382,442.33
6-Hydroxykaempferol-7-O-glucoside	464.10	C_21_H_20_O_12_	[M+H]^+^	Flavonoids	13,311,760.78
Quercetin-4’-O-glucoside	464.10	C_21_H_20_O_12_	[M-H]^−^	Flavonoids	12,717,017.62
Apigenin-8-C-Glucoside	432.11	C_21_H_20_O_10_	[M-H]^−^	Flavonoids	12,644,006.08
Quercetin-7-O-glucoside	464.10	C_21_H_20_O_12_	[M-H]^−^	Flavonoids	10,904,124.49
Hesperetin-5-O-glucoside	464.13	C_22_H_24_O_11_	[M-H]^−^	Flavonoids	10,868,954.34
Epicatechin	290.08	C_15_H_14_O_6_	[M+H]^+^	Flavonoids	10,284,549.50
Apigenin-6-C-(2″-glucosyl) arabinoside	564.15	C_26_H_28_O_14_	[M+H]^+^	Flavonoids	9,599,036.91
Kaempferol-3-O-neohesperidoside	594.16	C_27_H_30_O_15_	[M+H]^+^	Flavonoids	8,546,569.95
Luteolin-8-C-glucoside	448.10	C_21_H_20_O_11_	[M+H]^+^	Flavonoids	8,392,862.50
Kaempferol-3-O-glucoside-7-O-rhamnoside	594.16	C_27_H_30_O_15_	[M+H]^+^	Flavonoids	7,126,110.29
Apigenin-6-C-glucoside	432.11	C_21_H_20_O_10_	[M+H]^+^	Flavonoids	6,679,619.10
Luteolin-6-C-glucoside	448.10	C_21_H_20_O_11_	[M+H]^+^	Flavonoids	6,384,049.48
Kaempferol-3-O-robinobioside	594.16	C_27_H_30_O_15_	[M+H]^+^	Flavonoids	6,261,724.21
Kaempferol-3-O-galactoside	448.10	C_21_H_20_O_11_	[M-H]^−^	Flavonoids	4,834,661.33
Apigenin-8-C-(2″-glucosyl) arabinoside	564.15	C_26_H_28_O_14_	[M+H]^+^	Flavonoids	4,606,711.30
Kaempferol-3-O-mannoside	448.10	C_21_H_20_O_11_	[M+H]^+^	Flavonoids	2,381,539.37
Kaempferol-3-O-glucoside	448.10	C_21_H_20_O_11_	[M+H]^+^	Flavonoids	603,975.00
syringaresinol-4-O-β-D-glucopyranoside	580.22	C_28_H_36_O_13_	[M-H]^−^	Lignans and Coumarins	253,124.00
Sphondin	216.04	C_12_H_8_O_4_	[M+H]^+^	Lignans and Coumarins	4,143,763.49
Saikolignanoside D	520.19	C_26_H_32_O_11_	[M+H]^+^	Lignans and Coumarins	1,012,993.75
Indigoticoside A	684.26	C_32_H_44_O_16_	[M-H]^−^	Lignans and Coumarins	210,510.75
Dihydrosesamin	356.13	C_20_H_20_O_6_	[M+H]^+^	Lignans and Coumarins	107,039.90
Dihydrodehydrodiconiferyl alcohol-9-O-β-D-xylopyranoside	492.20	C_25_H_32_O_10_	[M+H]^+^	Lignans and Coumarins	120,019.25
Dihydrodehydrodiconiferyl alcohol-9-O-β-D-glucopyranoside	522.21	C_26_H_34_O_11_	[M-H]^−^	Lignans and Coumarins	256,625.75
coumarin	146.04	C_9_H_6_O_2_	[M+H]^+^	Lignans and Coumarins	830,636.75
Scopoletin (7-Hydroxy-6-methoxycoumarin)	192.04	C_10_H_8_O_4_	[M+H]^+^	Lignans and Coumarins	416,175.00
Psoralen	186.03	C_11_H_6_O_3_	[M+H]^+^	Lignans and Coumarins	34,230,637.64
Pinoresinol	358.14	C_20_H_22_O_6_	[M-H]^−^	Lignans and Coumarins	11,1167.86

**Table 3:** Regression equations for major phenolic compounds, R^2^ values, linear ranges, and limit of detection.

Phenolic Compounds	Regression Equations	R^2^	Linear Ranges (ng/mL)	Limit of Detection (mg/L)
Epicatechin	Y = 3215.72X + 104.38	0.9998	1.06–106	0.000529
(+)-Catechin	Y = 1921.19X	0.9997	2.16–216	0.000511
Luteolin 8-C-glucoside	Y = 28.48X + 18.88	0.9985	49.1–4905	0.000108
Rutin	Y = 65.94X + 992.05	0.9976	64.2–6415	0.00321
Hesperetin	Y = 38636.59X − 728.84	0.9981	0.078–7.8	0.00039
Caffeic acid	Y = 205.39X − 115.63	0.9996	4.5–450	0.000225
Ferulic acid	Y = 21.78X − 5.54	0.9984	5.82–582	0.000291
Chlorogenic acid	Y = 825.1X + 2121.19	0.9992	1.04–520	0.0000521
Gallic acid	Y = 26.16X − 105.28	0.9981	5.96–596	0.000298
Kaempferol	Y = 1.53X + 71.94	0.9998	53.7–5365	0.0268
Quercetin	Y = 157.65X − 67.62	0.9956	0.833–83.3	0.000416
Salicylic acid	Y = 143.1X + 96.53	0.9978	0.654–65.4	0.00245
Apigenin	Y = 28638.1X + 887.87	0.9988	0.071–7.13	0.00000356

**Table 4:** The contents of the main individual polyphenols of DYFFs (n = 3).

	Individual Polyphenols (mg/kg DW)							
	Epicatechin	(+)-Catechin	Luteolin 8-C-Glucoside	Rutin	Hesperetin	Caffeic Acid	Ferulic Acid	Chlorogenic Acid
ChCl-MaA	13.9 ± 0.4 ^e^	1.5 ± 0.1 ^e^	32.7 ± 1.1 ^e^	556 ± 14 ^g^	0.35 ± 0.017 ^d^	3.1 ± 0.05 ^e^	6.8 ± 0.4 ^f^	7007 ± 245 ^b^
ChCl-TA	15.0 ± 0.4 ^e^	1.3 ± 0.03 ^f^	34.1 ± 1.5 ^e^	567 ± 11 ^g^	0.39 ± 0.010 ^c^	3.3 ± 0.08 ^d^	8.9 ± 0.3 ^d^	7980 ± 290 ^a^
ChCl-Xyl	20.4 ± 0.8 ^d^	1.9 ± 0.07 ^c^	29.1 ± 1.6 ^f^	533 ± 7 ^h^	0.32 ± 0.012 ^e^	3.1 ± 0.04 ^e^	8.8 ± 0.4 ^d^	5489 ± 84 ^d^
80%ethanol	39.9 ± 0.5 ^a^	3.9 ± 0.05 ^a^	53.7 ± 2.2 ^a^	943 ± 7 ^a^	0.55 ± 0.013 ^a^	6.5 ± 0.1 ^a^	21.3 ± 0.8 ^a^	7511 ± 455 ^a^
ChCl-EtG	25.9 ± 0.6 ^b^	2.1 ± 0.03 ^b^	34.2 ± 0.9 ^e^	626 ± 15^e^	0.39 ± 0.005 ^c^	2.8 ± 0.1 ^f^	12.9 ± 0.7 ^b^	6179 ± 57 ^c^
ChCl-Urea	12.6 ± 0.4 ^f^	0.8 ± 0.05 ^h^	35.0 ± 2.8 ^d^	542 ± 24 ^g^	0.29 ± 0.010 ^f^	1.9 ± 0.1 ^h^	9.0 ± 0.5 ^d^	4751 ± 229 ^e^
ChCl-LaA	11.7 ± 0.1 ^g^	1.3 ± 0.05 ^f^	48.2 ± 0.7 ^b^	797 ± 9 ^b^	0.46 ± 0.006 ^b^	4.1 ± 0.08 ^b^	7.2 ± 0.2 ^e^	7824 ± 197 ^a^
ChCl-Glu	13.4 ± 0.3 ^f^	1.6 ± 0.07 ^d^	26.9 ± 1.02 ^g^	456 ± 4 ^i^	0.27 ± 0.004 ^g^	1.8 ± 0.04 ^i^	8.4 ± 0.2 ^d^	3734 ± 54 ^f^
Bet-MaA-Glu	10.7 ± 0.2 ^h^	1.0 ± 0.02 ^g^	38.5 ± 1.6 ^d^	606 ± 10 ^f^	0.32 ± 0.015 ^e^	4.0 ± 0.01 ^c^	7.5 ± 0.3 ^e^	6828 ± 203 ^b^
Bet-Gly	23.3 ± 0.1 ^c^	1.7 ± 0.07 ^d^	36.6 ± 0.7 ^d^	635 ± 2 ^e^	0.30 ± 0.009 ^f^	2.4 ± 0.07 ^g^	11.1 ± 0.4 ^c^	3923 ± 155 ^f^
Bet-LaA	11.4 ± 0.5 ^g^	1.4 ± 0.007 ^f^	50.3 ± 1.8 ^a^	739 ± 5 ^c^	0.35 ± 0.003 ^d^	4.0 ± 0.09 ^c^	6.3 ± 0.2 ^f^	5867 ± 276 ^c^
MA-EG	9.2 ± 0.3 ^i^	0.1 ± 0.009 ^g^	37.1 ± 1.6 ^d^	624 ± 15 ^e^	0.34 ± 0.017 ^d^	3.9 ± 0.1 ^c^	7.4 ± 0.3 ^e^	6187 ± 326 ^c^
Pro-LaA	3.5 ± 0.2 ^k^	0.2 ± 0.006 ^j^	47.8 ± 1.7 ^b^	743 ± 10 ^c^	0.33 ± 0.003 ^e^	4.3 ± 0.1 ^b^	6.2 ± 0.2 ^f^	5588 ± 205 ^c^
Pro-EtG	6.6 ± 0.3 ^j^	0.4 ± 0.009 ^i^	41.4 ± 2.4 ^c^	641 ± 28 ^d^	0.27 ± 0.008 ^g^	2.0 ± 0.03 ^h^	14.1 ± 0.69 ^b^	2720 ± 150 ^g^
Pro-MaA	3.7 ± 0.2 ^k^	0.2 ± 0.004 ^j^	43.8 ± 1.1 ^c^	637 ± 13 ^e^	0.27 ± 0.006 ^g^	3.5 ± 0.14 ^d^	7.7 ± 0.28 ^e^	5136 ± 218 ^d^

Note: a, b, c, etc., indicate differences (*p* < 0.05), and the results are expressed as the mean ± standard deviation of three values.

**Table 5:** The contents of the main individual polyphenols of DYFFs with different solvents.

Solvents	Inhibition of Pancreatic Lipase Activity (µg OTT/mL)	Inhibition of α-Amylase Activity (mg Acar/mL)
Bet-MaA-Glu	6009.75 ± 167.52 ^a^	0.024 ± 0.0042 ^j^
ChCl-LaA	5810.34 ± 483.24 ^a^	3.54 ± 0.23 ^b^
MA-EG	5171.41 ± 44.62 ^b^	1.09 ± 0.049 ^e^
Pro-LaA	3208.24 ± 117.44 ^c^	0.51 ± 0.045 ^f^
Pro-MaA	3144.42 ± 143.54 ^c^	0.015 ± 0.0029 ^k^
ChCl-TA	2873.93 ± 66.98 ^d^	0.094 ± 0.019 ^i^
ChCl-MaA	2668.59 ± 19.63 ^e^	2.93 ± 0.31 ^c^
Bet-LaA	1922.24 ± 126 ^f^	0.51 ± 0.20 ^f^
ChCl-EtG	1093.51 ± 148.23 ^g^	0.23 ± 0.027 ^g^
ChCl-Xyl	846.33 ± 105.93 ^h^	0.21 ± 0.0079 ^g^
ChCl-Glu	646.94 ± 85.00 ^i^	0.029 ± 0.0059 ^j^
Pro-EtG	604.65 ± 63.56 ^i^	0.13 ± 0.025 ^h^
ChCl-Urea	585.42 ± 30.4 ^i^	5.87 ± 0.80 ^a^
Bet-Gly	526.19 ± 13.88 ^j^	2.40 ± 0.25 ^d^
80% ethanol	413.89 ± 22.25 ^k^	0.23 ± 0.026 ^g^

Note: a, b, c, etc., indicate differences (*p* < 0.05), and the results are expressed as the mean ± standard deviation of three values.

**Table 6:** Scores of different extraction solvent systems based on PCA.

Observations	PC1	PC2	PC3
ChCl-MaA	−0.29546	−0.93459	−0.44063
ChCl-TA	0.01902	−0.99979	−0.45188
ChCl-Xyl	−0.21062	−0.32219	−1.63772
ChCl-EtG	0.42418	0.44115	−1.4058
ChCl-Urea	−0.69838	0.19857	−0.47194
ChCl-LaA	0.85558	−0.53284	0.40535
ChCl-Glu	−1.17432	1.05179	−1.04557
Bet-MaA-Glu	0.17668	−1.63425	−0.23221
Bet-Gly	−0.46866	1.73949	−0.00851
Bet-LaA	0.36246	0.03379	1.57327
Pro-LaA	−0.25843	0.0351	1.84561
Pro-EtG	−1.12726	1.41045	0.91118
Pro-MaA	−0.3579	−0.70581	0.39098
80% ethanol	3.02756	1.15562	−0.15378
MA-EG	−0.27445	−0.93647	0.72165

**Figure A1:**
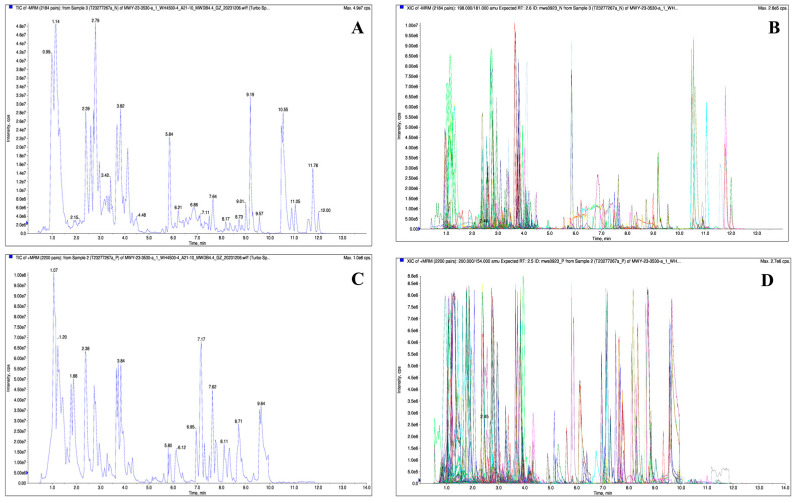
Total ions current in negative ion mode (**A**). Multimodal map of MRM metabolite detection in negative ion model (**B**). Total ions current in positive ion mode (**C**). Multimodal map of MRM metabolite detection in positive ion model (**D**).

## Data Availability

The raw data supporting the conclusions of this article will be made available by the authors on request.

## Appendix A

Total ions current (TIC) and a multimodal map of MRM metabolite detection (XIC) are shown in Figure A1A–D.
